# Immune Response Failure in Paucisymptomatic Long-Standing SARS-CoV-2 Spreaders

**DOI:** 10.3390/clinpract11010021

**Published:** 2021-03-01

**Authors:** Salvatore Corrao, Francesco Gervasi, Francesca Di Bernardo, Christiano Argano

**Affiliations:** 1 Department of Internal Medicine, COVID Unit, National Relevance and High Specialization Hospital Trust ARNAS Civico, Di Cristina, Benfratelli, 90127 Palermo, Italy; chargano@yahoo.it; 2 Dipartimento di Promozione della Salute, Materno Infantile, Medicina Interna e Specialistica di Eccellenza “G. D’Alessandro”, PROMISE, University of Palermo, 90127 Palermo, Italy; 3 Dipartimento di Medicina, UOC Medicina Interna iGR, ARNAS Civico, Di Cristina, Benfratelli, Piazza Nicola Leotta, 90127 Palermo, Italy; 4 Specialized Laboratory of Oncology, National Relevance and High Specialization Hospital Trust ARNAS Civico, Di Cristina, Benfratelli, 90127 Palermo, Italy; francesco.gervasi@arnascivico.it; 5 Department of Microbiology and Virology, National Relevance and High Specialization Hospital Trust ARNAS Civico, Di Cristina, Benfratelli, 90127 Palermo, Italy; francesca.dibernardo@arnascivico.it

**Keywords:** SARS-CoV-2, paucisymptomatic patients, multiparametric flow cytometry, immune system deficiency, COVID-19

## Abstract

The coronavirus disease 2019 (COVID-19), caused by severe acute respiratory syndrome coronavirus 2 (SARS-CoV-2), has rapidly spread throughout the world. This disease has a spectrum of different clinical pictures with different outcomes. Herein, we report all the data from three paucisymptomatic patients during a hospital stay that might represent a paradigmatic example of the method by which SARS-CoV-2 is shed. We demonstrated the lack of an adequate qualitative and quantitative immune response by multiparametric flow cytometry analysis. Our data can provide a new perspective about the method by which SARS-CoV-2 is shed and the clinical weight of viral persistence. In all three cases, the long persistence of the virus and the consistent reduction in both innate and adaptative immune cells are not associated with greater disease severity. These patients might represent at least part of the population. In particular, one patient oscillated between positive and negative swab tests several times without presenting any immune response. In all three cases, the immune response failure was not associated with a clinically significant involvement, indicating that it is not the virus’s ability to impair the immune system, as well as its presence and persistence the fundamental mechanism that might causally lead to death. Finally, this kind of immune response in paucisymptomatic patients could pose a considerable danger to public health that questions the quarantine period. It is urgent to quantify the phenomenon with a large sample study.

## 1. Introduction

In the last nine months, the world faced the COVID-19 pandemic, ranging from an asymptomatic and paucisymptomatic form to more critical conditions [1]. At the time of writing, there have been 32,867,270 confirmed cases, with 994,499 deaths [2]. Behaviour of the immune systems of asymptomatic and paucisymptomatic patients is crucial to understand how the virus works and how the infection can spread. An effective immune response against SARS-CoV-2 and other viruses depends on the activation of cytotoxic T cells. Recent studies showed the kinetics and breadth of the immune response in patients with mild to moderate COVID-19 [3], demonstrating damage of function of CD4^+^ T helper-cells that may predispose subjects to severe disease and exhaustion of CD8^+^ T cytotoxic lymphocytes [4] and Natural Killer (NK) [5] cells that may reduce the cellular immune response to SARS-CoV-2. We now present our experience with three patients that tested positive for SARS-CoV-2 and who were admitted to the COVID-19 Department of Internal Medicine of ARNAS-Civico in Palermo, characterized by a deficient immune response highlighted by MPFC, and that could explain a possible mechanism for the spreading of SARS-CoV-2 in asymptomatic and paucisymptomatic patients.

## 2. Patients

### 2.1. Case-Report 1

An 81-year-old woman, who tested positive for SARS-CoV-2 on March 22, was admitted from the nursing home on April 11 for suspected spontaneous prosthetic fracture of the left femur. On admission, the chest ultrasound showed diffuse bilateral B lines, and the chest computed tomography (CT) scan documented basal bilateral lung interstitiopathy. Treatment was started with hydroxychloroquine, azithromycin, and fondaparinux. On day 4 after admission, piperacillin/tazobactam was added for upper urinary tract infection. On day 7, 13, and 15, treatments with azithromycin, piperacillin/tazobactam, and hydroxychloroquine were stopped. Laboratory findings at admission showed mild normochromic normocytic anaemia (Hb 10.3 gr/dL) and leukopenia (WC 1990/µL) with severe lymphopenia (130/µL), elevated blood urea nitrogen (BUN), creatinine, fibrinogen, and D-Dimer levels (117 mg/dL, 1.4 mg/dL, 628 mg/dL, 1950 ng/mL, respectively). Starting on day 3, BUN and creatinine levels progressively increased, reaching a peak on day 7 (creatinine 2.48 mg/dL, BUN 141 mg/dL) returning to baseline on day 16. On day 4, leucopenia and lymphopenia resolved. PCR and procalcitonin levels began to rise, for concomitant urinary tract infection, reaching a peak on day 5 (PCR 5.5 mg/dL and procalcitonin 23.26 ng/dL) and were within the normal ranges on day 16. D-dimer levels were kept high throughout the hospital stay. Oxygen saturation (SaO2) ranged from 92 to 98% in room air.

The patient also underwent a SARS-CoV-2 RNA test through a nasopharyngeal swab on day 7 after admission (negative), day 8 (positive), day 13 (positive), day 22 (negative), day 24 (positive), day 26, 27, and 30 (all negative) and antibody screening on day 13, 24, 31 after admission (all negative). An MPFC analysis was carried out on day 10 and day 28 after admission. The patient is still hospitalized.

### 2.2. Case-Report 2

A 78-year-old man was admitted from a tertiary cardiovascular hospital on April 11. He underwent ascending aorta aneurysm’s replacement on March 24 and was positive based on a nasopharyngeal swab on April 8. Chest CT scan showed fibrotic striae in the anterior segment of the right upper lobe with bilateral pleural effusion and atelectasis of the adjacent pulmonary parenchyma, median sternotomy outcomes, and mild pericardial effusion.

Treatment was started with azithromycin and fondaparinux. Admission laboratory tests showed mild microcytic hypochromic anaemia (Hb 9.9 gr/dL) and mild leucopenia (WC 3.680/µL) with normal lymphocytes, elevated BUN, creatinine, fibrinogen, and D-Dimer levels (59 mg/dL, 1,73 mg/dL, 508 mg/dL, 5212 ng/mL, respectively) with somewhat elevated liver enzymes AST 50/ALT 57 IU and PCR and procalcitonin levels of 6.28 mg/dL and 0.17 ng/dL. On day 6, BUN, creatinine, white blood cells, and AST/ALT were within the normal range as well as PCR and procalcitonin on day 10. D-dimer levels kept a slower downward trend (on day 24, 2103 ng/dL). SaO_2_ ranged from 93 to 98% in room air. The patient also underwent nasopharyngeal swabs on day 10, 16, and 23 (all positive) and on day 39 and 40 (both negative) and antibody screening on day 5 (negative), on day 17 (IgM+, IgG+) and day 24 (doubtful for IgM, IgG+), and day 34 and 44 (IgM−, IgG+). MPFC assay was carried out on day 10 and day 35 after admission. The patient was discharged on May 21.

### 2.3. Case-Report 3

A 37-year-old man was admitted on April 13 because of chest pain. The patient tested positive for SARS-CoV-2 on March 2; he was in home isolation since that date. The chest CT scan showed some blurred parenchymal consolidations with a ground-glass appearance in the right upper lobe’s apical segment. On day 1, treatment with azithromycin and hydroxychloroquine was started. On day 5, azithromycin therapy was discontinued.

All haematochemical tests were within the normal range including Troponin-T values. SaO2 ranged from 95 to 98% in room air. The patient also underwent antibody screening on day 3 (IgM− and IgG−) and a nasopharyngeal swab on day 8 (positive). MPFC test was performed 7 days after admission. He was discharged on April 22 because he clinically healed; the nasopharyngeal swab was still positive.

## 3. Methods

### 3.1. Flow Cytometry

A flow cytometry six-eight-colour test covering 34 different subsets of immune cells from just 2 mL of human peripheral blood EDTA anticoagulated was developed [6]. The MPFC immunophenotyping test for direct staining of whole blood samples has been optimized. This technique allows the detection of all circulating immune cells and reduces the necessary flow cytometry preparation steps. The direct staining procedure minimizes the effort and variations in sample preparation. It represents a saving of time, an additional prerequisite for easy clinical application, which requires less than 20 min of reasonable time.

**Figure 1:**
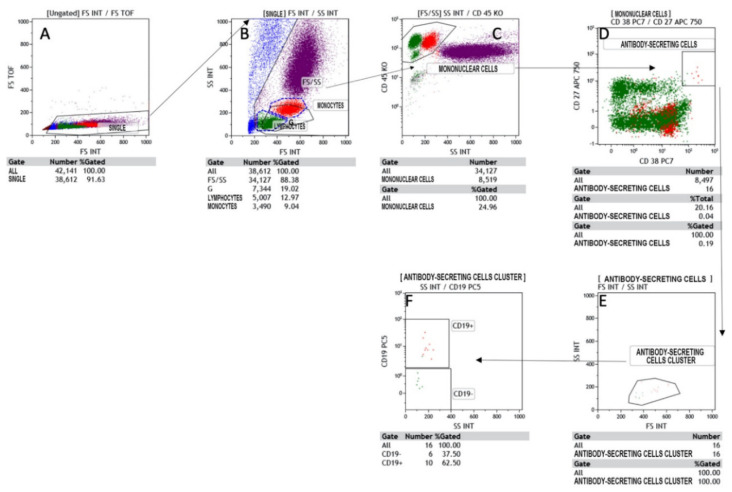
Flow cytometry gating strategy to identify the antibody-secreting cells (ASC) in Covid-19 patients. (**A**) shows the region SINGLETS, (**B**) gated on SINGLETS region display physical characteristic FS vs SS in which we are drawn lymphocytes and monocytes region, (**C**) gated by FS/SS region the intersection between CD45 vs SS identify the Mononuclear cells gate, (**D**) are gated by mononuclear cells. (**E**) and (**F**) are respectively characterized the cell cluster and the CD19 expression.

**Figure 2:**
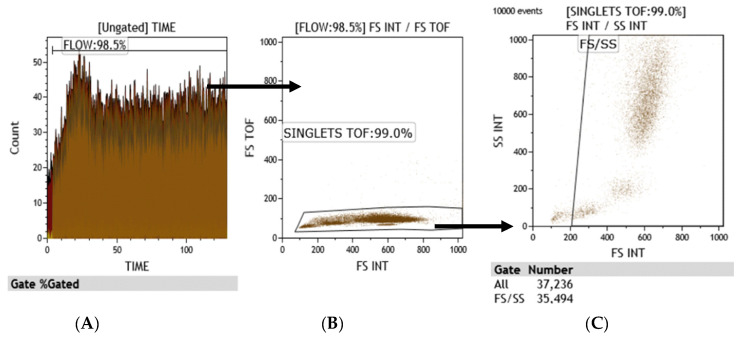
All cells-gate plays a central role in the identification of the subset cellular population of peripheral blood. All irregularities were excluded by the definition of Flow-gates (**A**); doublets were excluded by a singlets gate drawn in the central dot plot (**B**), forward scatter signal (FS) integral (INT) versus time of flight (TOF). Finally (**C**), all cells-gate was drawn on its FS vs. a side scatter (SS) characteristic. In this gate, the shifted events to the left, lower FS, were considered dead or dying cells and were removed from the analysis.

**Figure 3:**
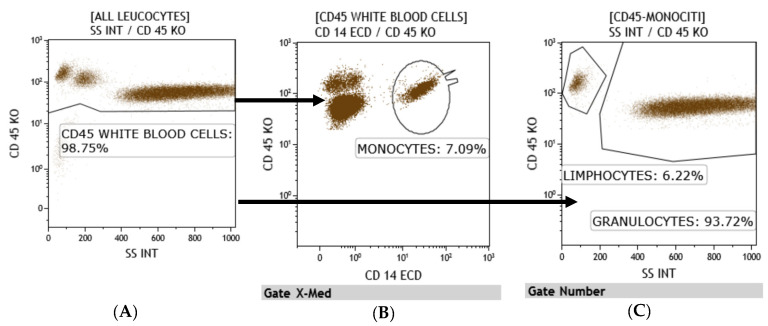
All cells-gate (**A**) can be subdivided based on its CD45 and SS characteristics into MONOCYTES (**B**), LYMPHOCYTES AND GRANULOCYTES (**C**), particularly dot plot C is gated on a boolean gate (all cell gate subtracted by monocytes). The percentage of the respective cellular population is shown in each dot plot.

**Figure 4:**
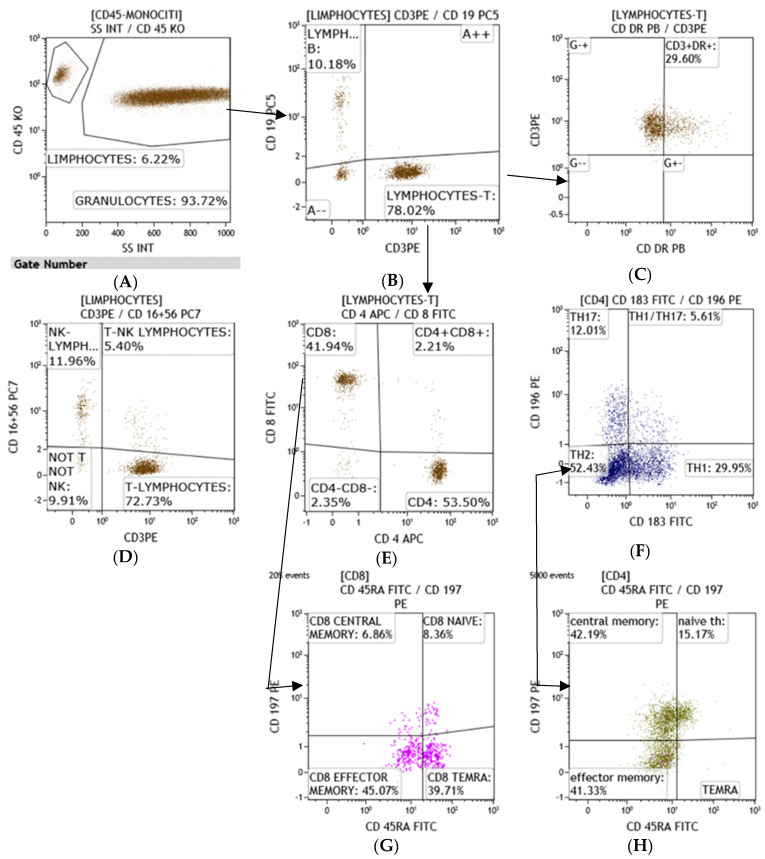
Gating strategy for the characterization of T, B, NK population and sixteen T cell subsets. Lymphocyte population was gated by CD45 expression (**A**); T lymphocytes are identified in (**B**) and T activated (CD3^+^DR^+^) are shown in (**C**); (**D**) displays T, NK and TNK lymphocytes gated on CD45^+^SSlin; T cell subset T-helper (CD3^+^CD4^+^), T-cytotoxic (CD3^+^CD8^+^) are gated by CD3 expression in (**E**); TH1, TH2, TH1/TH17, TH17 were gated by CD4 expression (**F**); Central memory (CM), naïve, Effector memory (EM), and EMRA were gated, by CD8 (**G**) or CD4 expression (**H**), respectively.

All lymphocyte populations and sub-populations T, T-helper, T-cytotoxic, Natural Killer, T/NK, and B were determined as a percentage and an absolute count. For this purpose, to allow detailed immunophenotyping of blood, a panel with monoclonal antibodies was designed: anti-CD45, anti-CD3, anti-CD5, anti-CD8, anti-CD16, anti-CD24, anti-CD25, anti-CD27, anti-CD38, anti-CD56, anti-CD20, anti-CD45RA, anti-CD183, anti-CD196, anti-CD197, anti-TCR -anti-TCR covering all major types of immune cells such as T and B cells, NK, monocytes, neutrophils, eosinophils and antibody-secreting plasma cells (CD38^++^CD27^++^CD19^±^) IgG class, and for phenotyping the T naïve-memory and T-helper 1, T-helper 2, T-helper 17 compartment (Figure 1, Figure 2, Figure 3 and Figure 4).

All monoclonal antibodies were obtained from Beckman–Coulter (Miami, FL, USA). Whole blood samples were incubated with monoclonal antibodies for 15 min at room temperature and were lysed with ammonium chloride for 20 min at 4 °C by a lyse-no wash method. At least 25,000 total events were acquired, excluding doublets and debris, on a Navios^TM^ Beckman–Coulter flow cytometer (Miami, FL, USA). The analysis of the obtained samples was carried out by Kaluza^TM^ analysis 2.1 software Beckman–Coulter (Miami, FL, USA), with gate SS/CD45 for the determination of lymphocyte populations and subpopulations and by double gate CD19/SS and CD38/SS for the determination of total and secreting plasma cells [7,8,9,10].

### 3.2. Microbiology

The molecular diagnostics of SARS-Cov-2 was carried out at the Laboratory of Virology of the Department of Microbiology, using the detection of the Single-Stranded Positive-Sense RNA Virus in rhino-pharyngeal swabs, by reverse transcription cDNA and polymerase chain reaction (RT-PCR) (Elitech Ingenius-Arrow SeGeneNimbus, Puteaux, France).

### 3.3. IgM and IgG Detection

A rapid qualitative membrane-based immunoassay method for detecting antibodies IgM and IgG to SARS-CoV2 was performed (Biosynex, Illkirch-Graffenstaden, France, COVID-19 BSS, IgM sensitivity 91.8%, specificity 99.2%; IgG sensitivity 100%, specificity 99.5%), using blood from a fingerstick.

## 4. Results

**Figure 5:**
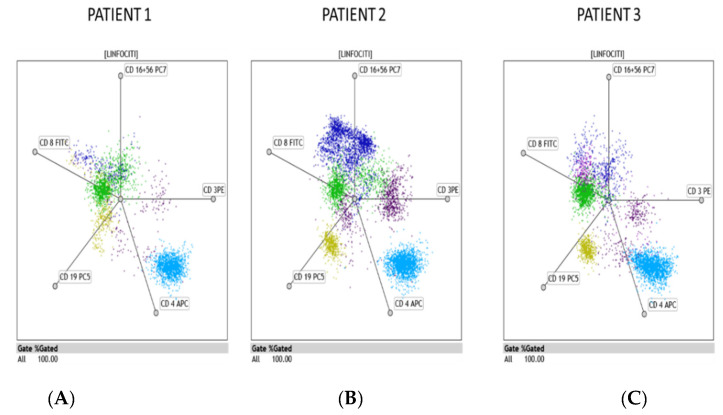
Distribution of the lymphocyte subpopulation and population in three covid-19 patients using the radar function of Kaluza analysis software (Beckam–Coulter). The figure shows the pattern distribution, in the electronic space, of the three major lymphocyte populations: T (CD3^+^) subdivided into CD4^+^ (T-helper) (cyan color) and CD8^+^ (T-cytotoxic) (green color), B (CD 19^+^) (ocra color), and NK (CD 16*CD 56*CD3^−^) (blue color) (**A**–**C**). It is of relevance that the difference in the TNK, NK, and B population practically disappeared in the first patient (**A**). In contrast, the second patient (**B**) showed a consistent presence of NK and B lymphocytes and the subdivision of the T-cytotoxic (CD3^+^CD8^+^) (green) population in two different populations occupying a well-defined space in the electronic dot plot (**C**).

Table 1 shows the clinical and laboratory characteristics of all subjects. Patient-1 had all cell populations below the normal range except for total plasma cells at first MPFC. T-helper, NK, B-cells, TNK, and ASCs were below the normal range 48 days after the first positive swab with a negative test for IgM and IgG 51 days after her first positive test alternative results from nasopharyngeal swabs (Table 2). Patient-2 also had total lymphocytes, T-helper, and NK-granzyme positive lower than the limits. The second MPFC showed the proportion of NK-granzyme and ASCs reverted to the normal range even if at the lower limits and a positive test for IgM and IgG 38 days after first nasopharyngeal swab positive (Table 2). A nasopharyngeal swab tested negative 42 days after the first positive test before discharge. Patient 3 had all cell populations within the normal range except for NK-granzyme at first MPFC. Patient 3 was transferred with nasopharyngeal test still positive. Figure 5 shows the distribution of the lymphocyte subpopulation and population patterns. It is worth outlining that all patients had the proportion of NK-granzyme positive (standard value > 50%) and antibody-secreting plasma cells (ASCs) below the normal range on day 30, 13, and 52 after the first positive swab before admission, with a negative test for IgM and IgG and nasopharyngeal swab positive. All three cases showed a total lack of anti-SARS-CoV2 IgG production thirty, thirteen, and fifty-two days after they tested positive.

## 5. Discussion

Recent studies showed a significant association between circulating lymphocytes and an inflammatory state with antibody-secreting plasma cell deficiency and the effectiveness of treatment in patients with COVID-19 [11,12]. In patients with severe COVID-19 disease, total lymphocytes and particularly T helper subsets, CD8 T-cells, and B-cells were significantly lower than in patients with a mild form. CD8 T-cells can be used as an independent predictor for COVID-19 severity and treatment efficacy. The exhaustion of immune cells measured by an increased expression of NKG2A (C-type lectin receptor with inhibitory effects) on NK and CD8^+^ could contribute to an insufficient immune response [5]. In NK cells, NKG2A leads to decreased expression of TNFα, IL-2, and IFNγ and reduced granzyme B levels [12]. Two cytokines, IL-6 and IL-10, highly present in SARS-CoV-2 infection [13], can reduce NK cell cytotoxicity [14]. In particular, IL-6 directly reduce the expression of perforin and granzyme B. In patients with COVID-19 admitted to the intensive care unit, an inverse correlation between serum levels of IL-6 and NK cell frequency expressing Granzyme A was found [15]. Kim et al. showed that asymptomatic patients might already be infectious because they had an initial viral load that might be a live virus [16]. Notably, in the asymptomatic carriers, the viral load was not high, and RT-PCR was negative 14 days after diagnosis in asymptomatic individuals. Another study found that the live virus could not be detected by culture in cases with a cycle threshold higher than 35 [17]. Park et al. showed that more than a quarter of subjects with mild symptoms admitted to a community treatment centre had negative results on secondary viral testing eight days after admission [18].

Our patients had no history of haematological or immune-system disease. The main finding in these three patients is the lack of adequate qualitative and quantitative immune responses. In the three cases, the MPFC showed a proportion of NK granzyme below the normal range and a number of ASCs equal to zero and one per microliter. It is well known the crucial role of antibody-secreting plasma cells for the rapid production of antibodies against ebolavirus [19] and the importance of T helper cells activated against influenza virus [20]. Our results are not in line with those of Theravadan and colleagues [3]. They found that antibody-secreting plasma cells appeared on day seven and peaked on day eight along with T helper cells and reached peak levels on day twenty during convalescence.

Moreover, our findings are not in accordance with previous ones [21] that showed IgM and IgG antibodies positive as early as the fourth day after illness onset and IgM and IgG seroconversion that increased from the ninth and eleventh day, respectively. Our findings agree with those of Varnaitė et al. [22], who found that in COVID-19 patients, an expansion of antibody-secreting plasma cells against SARS-CoV-2 occurred 19 days after COVID-19 symptom debut with a production of IgM and IgG, consistent with Lee et al. [23], who highlighted that in acute respiratory syncytial virus infection, antibody-secreting plasma cell expansion could be detected 22–45 days after the onset of symptoms due to the longer acute respiratory syncytial virus shedding time in the respiratory tract, indicating that the kinetics of the ASC response might be due to pathogen persistence. In our opinion, these three patients were paradigmatic of the true pathogenetic aim of SARS-CoV2 that tries to contaminate, remain, and spread as long as possible. In these patients, the ability of the virus to impair immune responses without a clinically significant involvement indicate that it is not the presence and the persistence of the virus that is the real mechanism that might causally lead to death. Based on these considerations, a hypothesis could be that the persistence of the virus along with a different higher or lower expression of receptor levels such as ACE2 in concert with the host’s membrane proteases TMPRSS2 [24], and CD-147 [25] together with the lack of an adequate immune response may lead to asymptomatic, mild, or the most severe form of the disease. As logical induction, this evidence could suggest a possible way of shedding of SARS-CoV2 by some patients that, labelled as recovered, could transmit the virus for weeks later because they did not have an adequate immune response. This is true, particularly in patient 1 that had been oscillating between negative and positive tests several times without any sign of immune response, attesting the full success of the virus. Patient 2 and 3 represent a partial success of SARS-CoV2 in impairing the individual immune system in any way that is useful for viral shedding.

## 6. Concluding Remarks

In conclusion, it is possible to speculate that the presented three patients might be representative of at least part of the paucisymptomatic population in which a failure of the innate immune system along with a deficiency of the acquired immune system does not allow patients to produce an adequate response with the absence of immunoglobulin production. These patients might still be long-standing spreaders without a clinically significant involvement. In our opinion, this kind of immune response in paucisymptomatic patients could lead to a massive danger to public health that questions the quarantine period. It is urgent to quantify the phenomenon with a large sample study.

## Figures and Tables

**Table 1:** Clinical and laboratory variables of the three patients.

	Patient-1	Patient-2	Patient-3
Sex	Woman	Man	Man
Age	81	78	37
Body Mass Index	18.3	23.8	26.1
Temperature	37.6	36.5	36.5
Systolic Blood Pressure _(mmHg)_	144	130	160
Diastolic Blood Pressure _(mmHg)_	76	55	100
Heart Rate _(bpm)_	76	88	105
Respiratory Rate _(breaths per minute)_	24	24	22
Oxygen Saturation	95	94	95
History of Smoking	-	Yes	-
Hypertension	Yes	Yes	Yes
Chronic Renal Failure	Yes	Yes	-
Heart Failure	-	Yes	-
Chronic Ischemic Heart Disease	-	Yes	-
Chronic Cerebrovascular Disease	-	Yes	-
Atrial Fibrillation	-	Yes	-
COPD	-	Yes	-
Dyslipidemia	-	Yes	-
Hydroxychloroquine	Yes	Yes	Yes
Azithromycin	Yes	Yes	Yes
LMWH	Yes	Yes	-
ACEi	Yes	-	Yes
Ca channel blockers	-	Yes	Yes
Beta-blockers	Yes	Yes	-
Loop diuretics	-	Yes	-
Aldosterone antagonists	-	Yes	-
Statins	-	Yes	-
Corticosteroid	-	-	-
DOC	-	-	-
Warfarin	-	-	-
Hb _(gr/dL)_	10.3	9.9	16.3
PLT _(×10_^3^_/µL)_	318	417	210
CRP _(mg/dL)_	1.48	6.28	0.07
PCT _(µg/L)_	0.24	0.17	0.05
Fibrinogen _(mg/dL)_	626	508	235
AST _(mg/dL)_	11	57	24
ALT _(mg/dL)_	10	50	40
D-Dimer _(ng/mL)_	1950	5212	433
Creatinine _(mg/dL)_	1.40	1.73	1.03
Blood Urea Nitrogen _(mg/dL)_	117	59	36
Na _(mmol/L)_	139	139	139
K _(mmol/L)_	4.50	3.79	4.02
Fasting Blood Sugar _(mg/dL)_	136	81	76

COPD (Chronic Obstructive Pulmonary Disease), LMWH (Low-Molecular-Weight Heparin), DOC (Direct Oral AntiCoagulants), ACEi (Angiotensin Converting Enzyme inhibitors), Hb (Hemoglobin), PLT (Platelets), CRP (C-Reactive Protein), PCT (Procalcitonin), AST (aspartate aminotransferase), ALT (alanine aminotransferase), Na (Sodium), K (Potassium), NK (Natural Killer), TNK (T Natural Killer).

**Table 2:** Flow cytometry variables of the three patients.

	Patient-1	Patient-2	Patient-3
Time from positive test and first flow cytometry _(days)_	30	13	52
Lymphocyte T (total n) (microL) _(normal range 1200–4000)_	380	620	1840
Lymphocyte T Helper (n) _(normal range 500–2000)_	190	400	1020
cytotoxic Lymphocyte T (n) _(normal range 200–1200)_	150	430	810
NK (total n) _(normal range 100–1200)_	50	360	220
Granzyme Nk > 50%	15	30	42
TNK _(normal range 100–500)_	40	50	140
Lymphocyte B (tot n) _(normal range 60–800)_	30	120	250
Plasma cells (tot n) _(normal range 1–11)_	7	2	5
Plasmacells (tot%) _(normal range 0.7–4.8)_	25	1.8	2.6
Antibody-secreting plasma cells (n) _(normal range 1–5)_	0	0	1
IgM	negative	negative	negative
IgG	negative	negative	negative
Time from positive test and second flow cytometry _(days)_	48	38	
Lymphocyte T (total n) (microL) _(normal range 1200–4000)_	850	740	
Lymphocyte T Helper (n) _(normal range 500–2000)_	480	400	
cytotoxic Lymphocyte T (n) _(normal range 200–1200)_	250	210	
NK (total n) _(normal range 100–1200)_	9	270	
Granzyme Nk > 50%	82	51	
TNK _(normal range 100–500)_	9	100	
Lymphocyte B (tot n) _(normal range 60–800)_	10	100	
Plasma cells (tot n) _(normal range 1–11)_	1	2	
Plasmacells (tot%) _(normal range 0.7–4.8)_	1.8	1.8	
Antibody-secreting plasma cells (n) _(normal range 1–5)_	0	1	
Time from positive test and first flow cytometry _(days)_	48	38	
IgM	negative	positive	
IgG	negative	positive	
